# De-Escalation by Reversing the Escalation with a Stronger Synergistic Package of Contact Tracing, Quarantine, Isolation and Personal Protection: Feasibility of Preventing a COVID-19 Rebound in Ontario, Canada, as a Case Study

**DOI:** 10.3390/biology9050100

**Published:** 2020-05-16

**Authors:** Biao Tang, Francesca Scarabel, Nicola Luigi Bragazzi, Zachary McCarthy, Michael Glazer, Yanyu Xiao, Jane M. Heffernan, Ali Asgary, Nicholas Hume Ogden, Jianhong Wu

**Affiliations:** 1 Laboratory for Industrial and Applied Mathematics (LIAM), Department of Mathematics and Statistics, York University, Toronto, ON M3J 1P3, Canada; btang66@yorku.ca (B.T.); scarabel@yorku.ca (F.S.); bragazzi@yorku.ca (N.L.B.); zjm@yorku.ca (Z.M.); mglazer@yorku.ca (M.G.); jmheffer@yorku.ca (J.M.H.); 2 The Interdisciplinary Research Center for Mathematics and Life Sciences, Xi’an Jiaotong University, Xi’an 710049, China; 3 CDLab—Computational Dynamics Laboratory, Department of Mathematics, Computer Science and Physics, University of Udine, 33100 Udine, Italy; 4 Department of Mathematical Sciences, University of Cincinnati, Cincinnati, OH 45221-0025, USA; xiaoyu@ucmail.uc.edu; 5 Modelling Infection and Immunity Lab, Centre for Disease Modelling, Department of Mathematics & Statistics, York University, Toronto, ON M3J 1P3, Canada; 6 Disaster & Emergency Management, School of Administrative Studies & Advanced Disaster & Emergency Rapid-response Simulation (ADERSIM), York University, Toronto, ON M3J 1P3, Canada; asgary@yorku.ca; 7 Public Health Risk Sciences Division, National Microbiology Laboratory, Public Health Agency of Canada, St. Hyacinthe, QC J2S 2M2, Canada; nicholas.ogden@canada.ca

**Keywords:** COVID-19, pandemics, physical and social distancing relaxation, reopening, mathematical model

## Abstract

Since the beginning of the COVID-19 pandemic, most Canadian provinces have gone through four distinct phases of social distancing and enhanced testing. A transmission dynamics model fitted to the cumulative case time series data permits us to estimate the effectiveness of interventions implemented in terms of the contact rate, probability of transmission per contact, proportion of isolated contacts, and detection rate. This allows us to calculate the control reproduction number during different phases (which gradually decreased to less than one). From this, we derive the necessary conditions in terms of enhanced social distancing, personal protection, contact tracing, quarantine/isolation strength at each escalation phase for the disease control to avoid a rebound. From this, we quantify the conditions needed to prevent epidemic rebound during de-escalation by simply reversing the escalation process.

## 1. Introduction

“Coronavirus disease 2019” (COVID-19) is a generally mild but sometimes severe and life-threatening infection caused by an emerging coronavirus, known as “Severe Acute Respiratory Syndrome Coronavirus 2” (SARS-CoV-2). The principle target molecule for entry of SARS-CoV2 is the angiotensin-converting enzyme 2 (ACE2). During phase 1 (“viral entry”), the virus passes all usual barriers like mucous and cilia and can get into alveolus via ACE2 as entry receptor. The alveolar barrier is very close to blood vessels, being less than 2 μm but the virus does not go into blood. The first phase is characterized by the ability of the virus to get down and dock alveoli. During phase 2 (“alveolar innate immunity”), type 2 pneumocyte type 1 interferon (IFN) response is weakened. In addition, resident alveolar macrophages that are actually in the alveolus space and are involved in phagocytosis are weakened too. During phase 3, macrophages from outside and neutrophils are recruited. These are in interstitium and also produce and release cytokines. Antigen presenting cells (APCs) are then activated and head off. During phase 4, T-cell responses start after a week with activation of CD8+ cytotoxic and CD4+ T cells. High amounts of IFN-gamma are produced at this stage. Humoral immunity is activated as well with IgM, IgG and IgA being massively released. Due to killing of lymphocytes, their effects are gradually reduced. During phase 5, usually the virus is being cleared. Failure of such a clearance leads to pulmonary remodeling, acute respiratory distress syndrome (ARDS), fibrosis and, eventually, death. Finally, during phase 6, in survivors, humoral and cellular immunity are established (even though the extent of such an establishment is yet to be elucidated in detail).

Since late December 2019, SARS-CoV-2 has quickly spread out from its first reported epicenter, city of Wuhan, Province of Hubei, People’s Republic of China, becoming a pandemic [1,2,3].

In Canada, the first COVID-19 case was reported on 15 January 2020 [4]. During the COVID-19 pandemic, most Canadian provinces have gone through four distinct phases of social distancing escalation:

**Escalation-Phase** **0**: Monitoring and international travel advisories;
**Escalation-Phase** **1**: School closure;
**Escalation-Phase** **2**: Emergency declaration, with closure of public events and recreational venues;
**Escalation-Phase** **3**: Closure of all non-essential workplaces.

Each province has had its own approach, with each phase beginning and ending at different dates. Within the escalation phases, different provinces have also declared a “state of emergency” or “state of public health emergency” on different dates, ranging from March 13 in Quebec to March 22 in Nova Scotia. With the declaration of emergency in place, provinces started to respond to the pandemic by closing all non-essential workplaces, providing a list of essential and non-essential businesses. Table 1 summarizes the main dates of interventions of the Canadian provinces.

In the present article, we will focus on the province of Ontario, as case study. In this province, the escalation phases were initiated, respectively, on March 14 (school closure), March 17 (emergency declaration with closure of public events and recreational venues, effective from March 18), and March 24 (closure of non-essential workplaces). More precisely, using a ministerial order, on March 12, the Ontario Minister of Education announced that all publicly funded schools across the province would be closed for two weeks following March Break. On March 17, Ontario declared a provincial state of emergency. The declaration imposed the closure of all bars and restaurants (except to the extent that such facilities provide takeout and food delivery), all facilities providing indoor recreational programs, all public libraries, all private schools, all licensed childcare centers, all movie cinemas, all theatres, including those offering live performances of music, dance and other art forms, and all concert venues. The Government of Ontario also prohibited all organized public events of over 50 people, including parades, and events or communal services within places of worship. The current state of emergency is effective until 6 May 2020. On March 18, Ontario and the USA agreed to restrict all non-essential travel across the shared border. In order to further reduce the contact within the Ontario population and mitigate COVID-19 spread, on March 23 the government of Ontario ordered all non-essential businesses to be closed for 14 days (effective March 24 at 11:59 pm) which was extended later to May 6. A full list of establishments that can remain open was provided on March 24 [5,6]. The list of non-essential businesses was gradually expanded in this period. For example, on 4 April 2020 only pharmacies, or businesses that primarily sell food and beverages were allowed to remain open. Finally, public school closures were extended a few times, with a current end date of 31 May 2020 (at the time of writing, 30 April 2020).

## 2. Materials and Methods

### 2.1. The Transmission Dynamics Model

**Figure 1:**
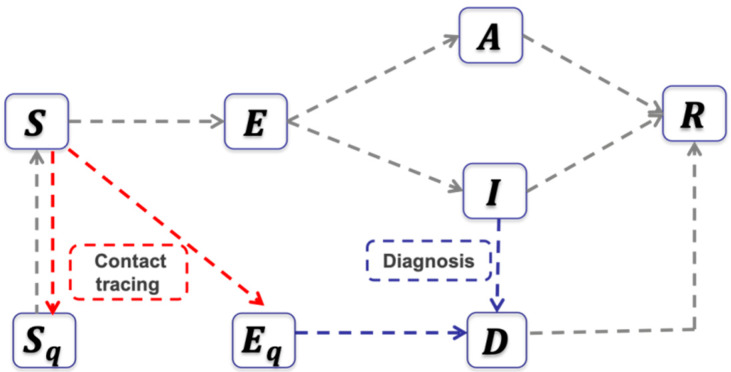
The flowchart of the transmission dynamics model, where the population is stratified by susceptible, exposed, asymptomatic infectious, symptomatic infectious, and recovered status, and by quarantine and isolation status.

We use the modelling framework of COVID-19 transmission developed in our previous studies [7,8] to model the effects of the comprehensive stringent package of public health measures (travel restrictions and intensive contact tracing followed by quarantine, isolation and lockdown of the Hubei province) implemented and enforced by the Chinese authorities. Here, we aimed to describe the COVID-19 transmission dynamics in Ontario, Canada. The population is divided into susceptible (S), exposed (E), asymptomatic infectious (A), infectious with symptoms (I), and recovered (R) compartments according to the epidemiological status of individuals. We also include diagnosed and isolated (D), isolated susceptible ($S_{q}$), and isolated exposed ($E_{q}$) compartments based on control interventions. Within the modelling framework, we also account for contact tracing, where a proportion, q, of individuals exposed to the virus are traced and isolated (we will also refer to q as ‘quarantine proportion’ or ‘quarantine fraction’). The quarantined individuals can either move to the compartment $E_{q}$ or $S_{q}$, depending on whether transmission occurred (with probability β), while the other proportion, 1−q, consists of individuals exposed to the virus who are missed from contact tracing and, therefore, move to the exposed compartment E once infected, or stay in the compartment S otherwise. The transmission dynamics model is

$$\begin{array}{l} S^{\prime }=-\left(\beta c+cq\left(1-\beta \right)\right)S\left(I+\theta A\right)/N+\lambda S_{q},\\ E^{\prime }=\beta c(1-q)S(I+\theta A)/N-\sigma E,\\ I^{\prime }=\sigma \varrho E-(\delta_{I}+\alpha +\gamma_{I})I,\\ A^{\prime }=\sigma (1-\varrho )E-\gamma_{A}A,\\ {S_{q}}^{\prime }=(1-\beta )cqS(I+\theta A)/N-\lambda S_{q},\\ {E_{q}}^{\prime }=\beta cqS(I+\theta A)/N-\delta_{q}E_{q},\\ D^{\prime }=\delta_{I}I+\delta_{q}E_{q}-(\alpha +\gamma_{D})D,\\ R^{\prime }=\gamma_{I}I+\gamma_{A}A+\gamma_{D}D, \end{array}$$

where N denotes the total population in Ontario. All model parameters are defined in Table 2. The corresponding flowchart is shown in Figure 1.

The control reproduction number of the transmission model with control interventions is given by

$$R_{C}=\frac{\beta \rho c\left(1-q\right)}{\delta_{I}+\alpha +\gamma_{I}}+\frac{\beta c\theta \left(1-\rho \right)\left(1-q\right)}{\gamma_{A}}$$

Since March 24, the province of Ontario has taken some additional public health measures to include the closure of all non-essential business and the improvement of testing to increase the detection rate. A more appropriate way to reflect these two major measures is to adopt a non-autonomous transmission dynamics model with a time-dependent contact rate and quarantine proportion. Specifically, after March 24 (corresponding to $T_{s}=27$ in our simulations), we assume that the contact rate c(t) and quarantine proportion q(t) are exponentially decreasing or increasing with exponential rates $r_{1}$ and $r_{2}$, respectively. That is, by introducing $T_{0},\;T_{1}$ and $T_{s}$ corresponding to March 14, March 18 and March 24, respectively, we define

$$c\left(t\right)=\{\begin{array}{c} c_{0},\;t<T_{0},\\ c_{1},\;t<T_{1},\\ c_{2},\;t<T_{s},\\ \left(c_{2}-c_{b}\right)e^{-r_{1}\left(t-T_{s}\right)}+c_{b},\;t\geq T_{s}, \end{array}$$

and

$$q\left(t\right)=\{\begin{array}{c} q_{0},\;t<T_{s},\\ \left(q_{0}-q_{b}\right)e^{-r_{2}\left(t-T_{s}\right)}+q_{b},\;t\geq T_{s}, \end{array}$$

where $c_{0},\;c_{1},\;c_{2}$ are the constant contact rates and $q_{0}$ the quarantine proportion before March 24. The parameters $c_{b}$ and $q_{b}$ denote the minimum contact rate and the maximum quarantine proportion, respectively, estimated after March 24. In this case, the effective reproduction number is time-dependent and given by

$$R_{t}=\frac{\beta \varrho c\left(t\right)\left(1-q\left(t\right)\right)}{\delta_{I}+\alpha +\gamma_{I}}+\frac{\beta c\left(t\right)\theta \left(1-\varrho \right)\left(1-q\left(t\right)\right)}{\gamma_{A}}.$$

### 2.2. Data

We obtain the data of the cumulative reported COVID-19 infected cases in Ontario, Canada, from the Government of Canada [5,6]. The data were released and analyzed anonymously.

### 2.3. The Parameter Identification and Estimation of Intervention Effectiveness in Different Escalation Phases

To reflect the different escalation phases in Ontario, we assume piecewise constant rates for the periods February 26–March 14 and March 14–18. During the period March 14–18, we keep all parameters as those for the period February 26–March 14; but, allow only the contact rate to change. After March 24, we assume an exponentially decreasing contact rate and an exponentially increasing quarantine rate. Further, to proceed with the parameter estimation, we fix the susceptible population as the total population of Ontario, which is around $1.471\times 10^{7}$. The value of the incubation period, the disease-induced death rate, the recovery rate of the diagnosed population, and the rate at which the quarantined uninfected contacts were released into the wider community are taken from published literature, as shown in Table 2 [7,8,9].

**Figure 2:**
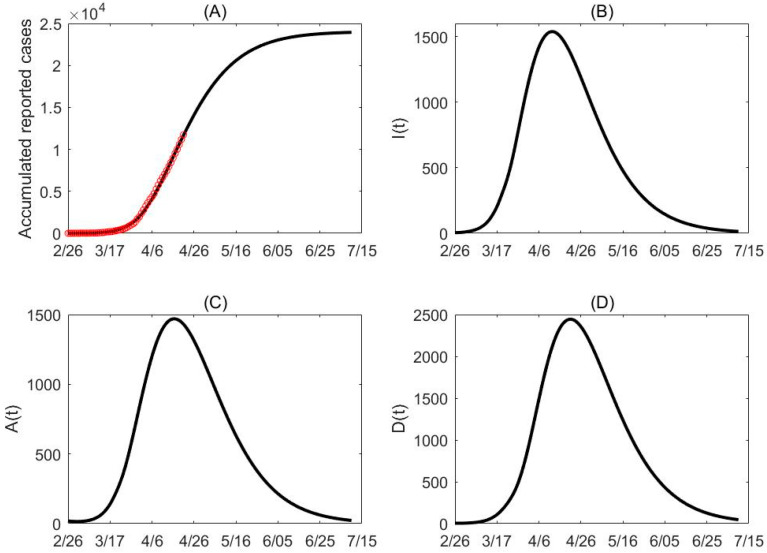
Data fitting and projection. (**A**) The red circles represent the cumulative confirmed cases in Ontario, Canada. (**B**–**D**) Based on the mean curve of the 500 times estimation, the population of I(t), A(t) and D(t) reach their peak times around April 12, April 16 and April 21 while the peak values are around 1538, 1469 and 2444, respectively. The final cumulative confirmed cases projected by the model is about 23,940.

Parameters for all phases are estimated together in a single model fit, by fitting all data from February 26 to April 21, using the least square method. By assuming the number of confirmed cases follows a Poisson distribution with mean given by the reported number, we generated 500 cumulative incidence data sets and estimated model parameters for each set. Consequently, the estimated mean values and their standard deviations of the parameters are provided in Table 2. The fitting results are shown in Figure 2A with the mean of the 500 fitting results being marked as black. Note that these intervention measures act synergistically, so these measures reflect the combined effectiveness of the interventions implemented during each phase. The code used is reported in Supplementary Materials S1.

**Figure 3:**
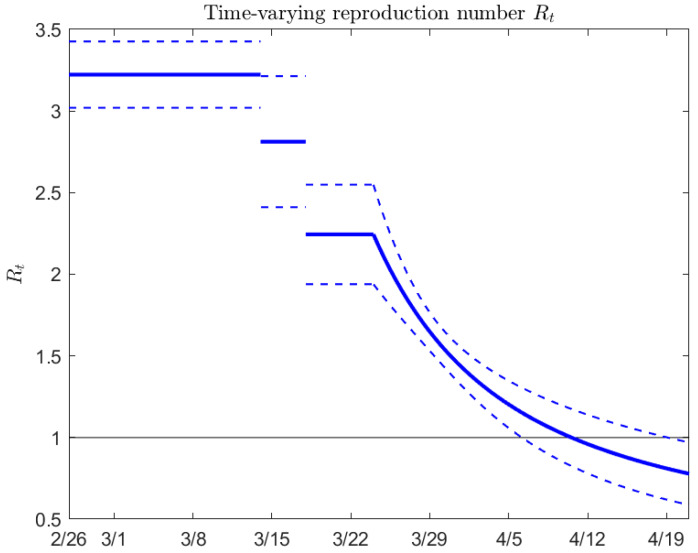
Estimated effective reproduction number $R_{t}$ in Ontario, Canada as a function of time (mean value and 95% confidence interval).

Figure 3 shows the estimated effective reproduction number $R_{t}$ as a function of time, from February 26 to April 21. Note that the average estimated reproduction number falls below one around April 11, with a 95% confidence interval from about April 6 to April 20.

## 3. De-Escalation Considerations

We now consider de-escalation using the same model. Using as baseline parameter values those estimated in the escalation phases (school closure to emergency declaration, March 14–18; emergency declaration to closure of non-essential workplaces, March 18–24; and closure of non-essential workplaces, after March 24), we investigate the feasibility of a de-escalation process that ‘reverses’ the escalation steps. In this sense, we consider three de-escalation phases:

**De-escalation-Phase** **1:** Opening of workplaces;
**De-escalation-Phase** **2:** Resumption of public events and activities;
**De-escalation-Phase** **3:** School opening.

Starting from baseline parameters as estimated from the escalation process (see Table 2), for each de-escalation phase we investigate the influence of parameter values on the effective reproduction number $R_{c}$. We consider, in particular, variations in the quarantine fraction, the time to diagnosis, the transmission probability, and the contact rates, in order to investigate which public health interventions (in terms of improved contact tracing, diagnosis, and further recommendations for usage of personal protective equipment like surgical masks) should be put in place in order to keep $R_{c}$ below 1.

We consider baseline scenarios corresponding to the contact rate estimated in the four different escalation phases, and under different scenarios where the contact rate is reduced (which can be reached by allowing only some people to resume regular activity, or by limiting the amount of time people are allowed to spend outside, or by rotating the working force among different working days plus longer weekend). The goal is to understand what combinations of parameters maintain $R_{c}<1$, and thus, avoid a rebound.

**Figure 4:**
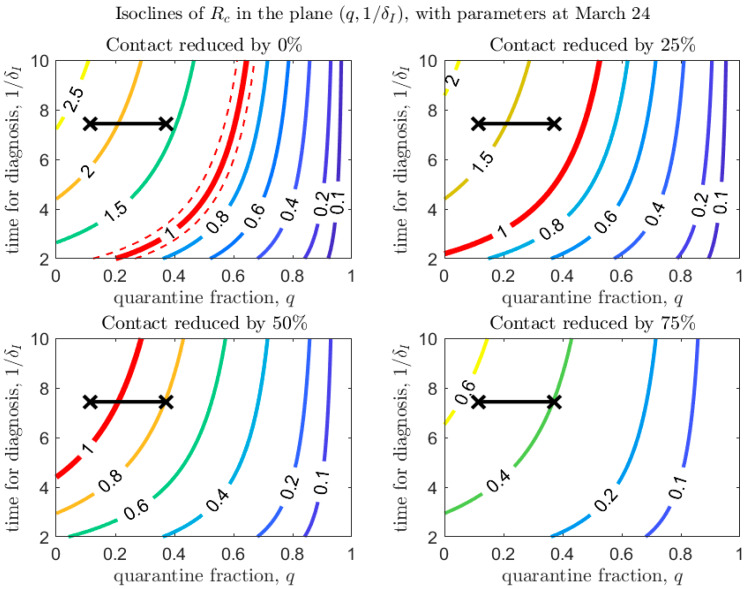
Values of $R_{c}$ in the plane $\left(q,1/\delta_{I}\right)$, with baseline parameters estimated on March 24 (top-left panel), and under different scenarios of contact rate reduction (other panels). The red line corresponds to $R_{c}=1$ (with dashed lines showing confidence intervals in the baseline scenario). The black crosses correspond to the parameters (max/min) estimated after March 24 (last day of the estimation period is April 21).

**Figure 5:**
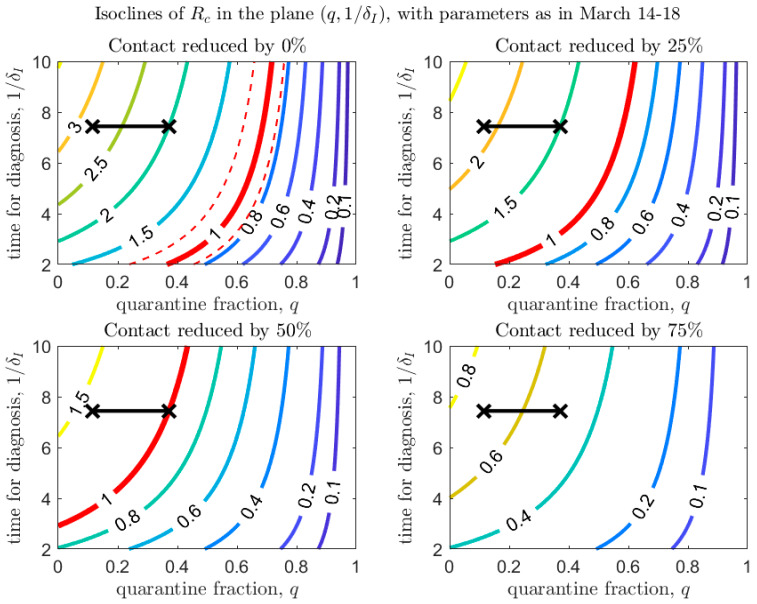
Same as Figure 4, with baseline parameters estimated in the period March 14–18.

**Figure 6:**
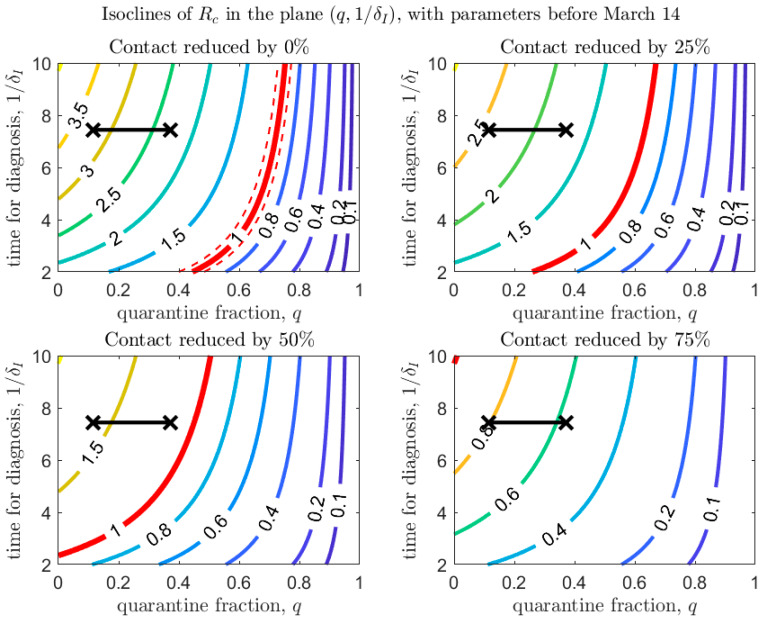
Same as Figure 4, with baseline parameters estimated in the period before March 14.

**Figure 7:**
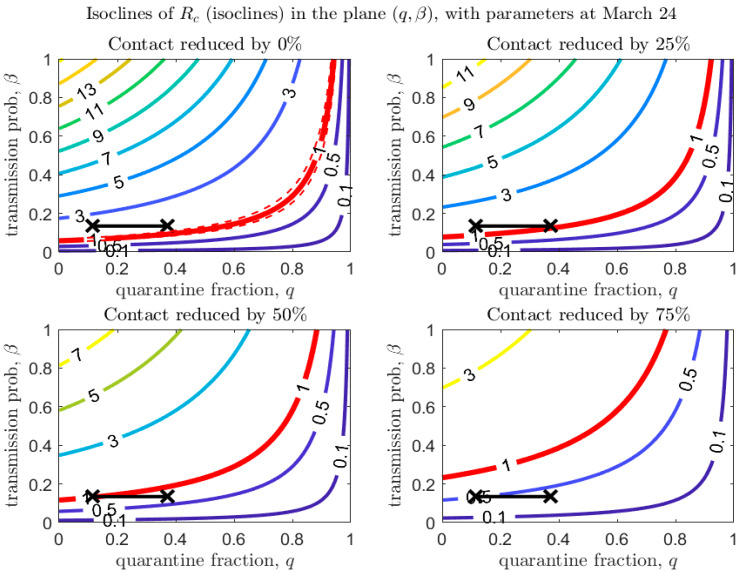
Values of $R_{c}$ in the plane (q,β), with baseline parameters estimated on March 24 (top-left panel), and under different scenarios of contact rate reduction (other panels). The red line corresponds to $R_{c}=1$ (with dashed lines showing confidence intervals in the baseline scenario). The black crosses correspond to the parameters (max/min) estimated after March 24 (last day of the estimation period is April 21).

**Figure 8:**
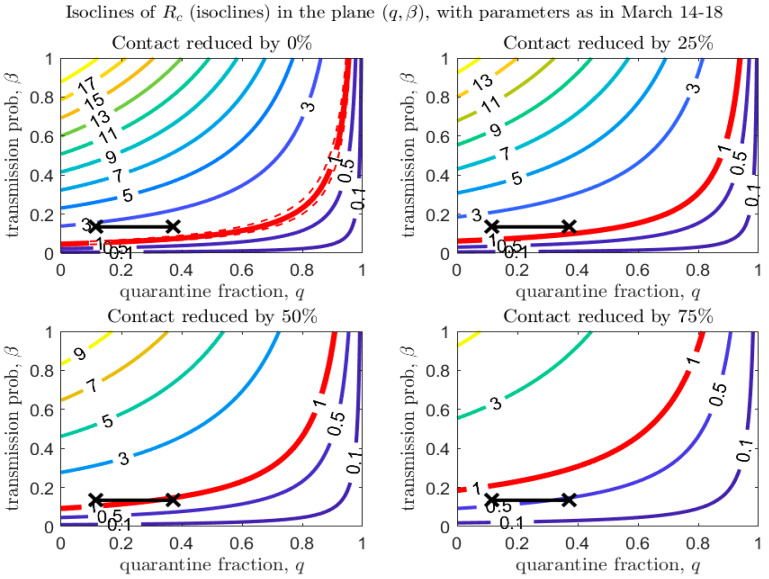
Same as Figure 7, with baseline parameters estimated in the period March 14–18.

**Figure 9:**
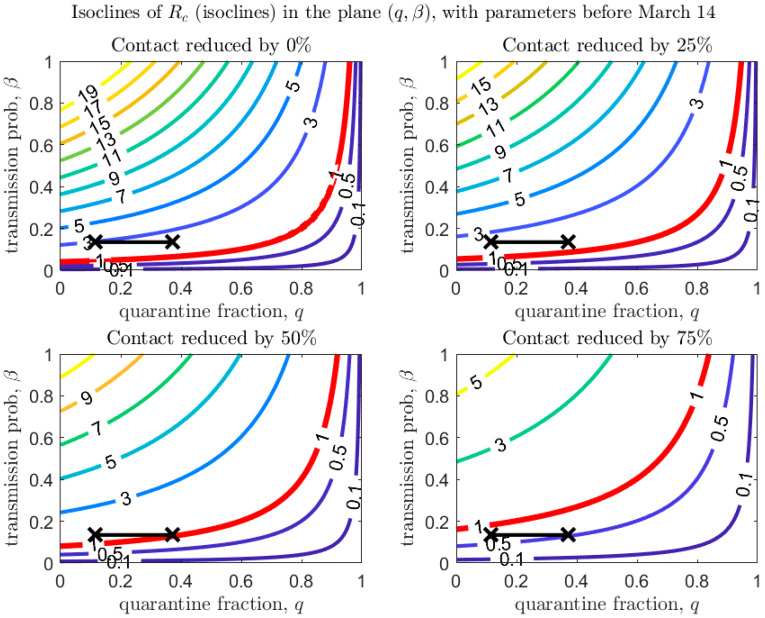
Same as Figure 7, with baseline parameters estimated in the period before March 14.

Figure 4, Figure 5 and Figure 6 show how $R_{c}$ depends on the quarantine fraction q and the time for diagnosis of a symptomatic individual, $1/\delta_{I}$. Figure 7, Figure 8 and Figure 9 show the dependence on q and on the transmission probability β. The figures refer to the three proposed de-escalation phases.

In the first de-escalation phase (Figure 4 and Figure 7) the baseline parameters are those estimated in the period March 18–24 (corresponding to the escalation Phase 2). In Figure 4 we observe that, if the current time for diagnosis is maintained, $R_{c}<1$ only if almost 60% of exposed contacts can be effectively traced and isolated (Figure 4, top-left panel). However, we also see that if some level of social distancing is maintained, that counters the decrease in the quarantine fraction, $R_{c}$ can still maintain a value less than unity. Additionally, we see that at the maximal quarantine capacity estimated after March 24 (parameter $q_{b}$ in Table 2), only a reduction in the (infection) contact rate between 25% and 50% maintains $R_{c}<1$. Finally, Figure 4 also shows that an improvement in the diagnosis rate has only a limited effect on the reduction in $R_{c}$, unless it is combined with improved isolation and quarantine efforts. A reduction in transmission probability also allows reductions in $R_{c}$ (Figure 7).

A similar analysis can be done for the second de-escalation phase, analyzed in Figure 5 and Figure 8.

Figure 6 and Figure 9 refer to the third de-escalation phase. In this case it is again interesting to note that, assuming the same diagnosis rate and no reduction in the contact rate, only unrealistically high values of the quarantine proportion (isolation of about 70% of exposed contacts) would avoid a rebound. With contact tracing of about one half of exposed contacts (q=0.5), a reduction in contact by only 50% would keep $R_{c}<1$ (Figure 6, bottom-left panel). A reduction in the (infection) contact rate or transmission probability (Figure 9) through strict usage of masks or other measures of social distancing seems unavoidable.

We stress that Figure 7, Figure 8 and Figure 9 also show that even a small reduction in the transmission probability can have a great impact in reducing the value of $R_{c}$, emphasizing the importance of masks and personal protective equipment.

**Figure 10:**
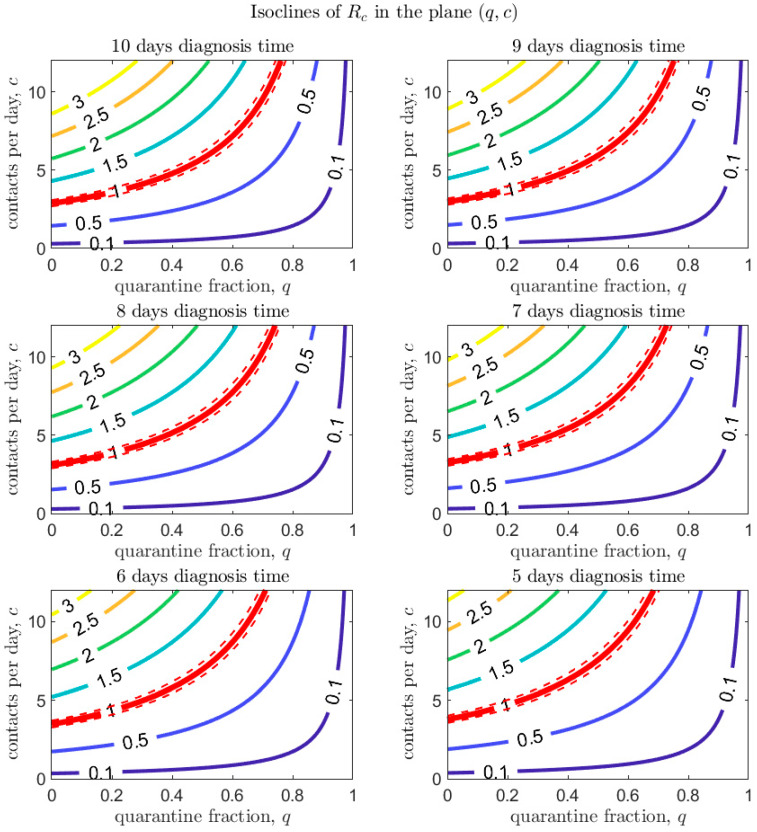
Values of $R_{c}$ in the plane (q,c) for different values of the time for diagnosis ($1/\delta_{I}$). The red line corresponds to $R_{c}=1$ (with dashed lines showing confidence intervals).

Finally, Figure 10 investigates the combined effect of quarantine fraction and contact rate on $R_{c}$, for given values of the time for diagnosis. Improving diagnosis seems to have a limited effect on reducing $R_{c}$.

Figure 10 investigates the combined effect of quarantine fraction and contact rate on $R_{c}$, for given values of the time to diagnosis. Both aspects, reduction in contact rate and improved contact tracing, seem to have relevant effect in control of the epidemic.

**Figure 11:**
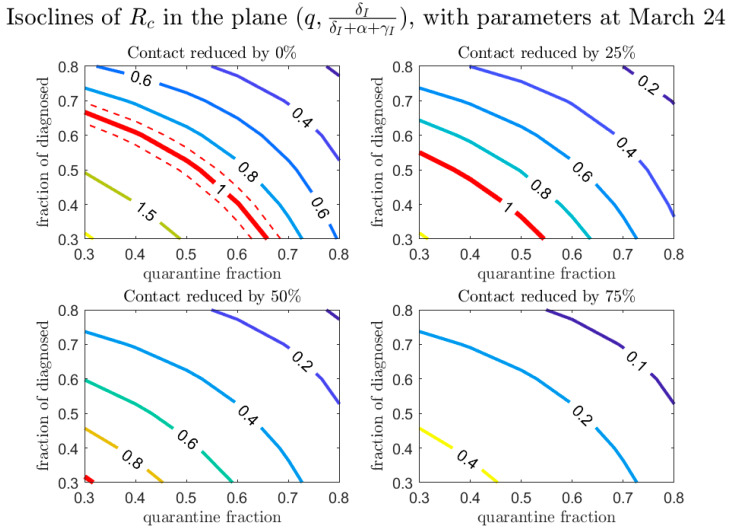
Values of $R_{c}$ in the plane $\left(q,\delta_{I}/(\delta_{I}+\alpha +\gamma_{I}\right))$, for different values of the contact rate. The red line corresponds to $R_{c}=1$ (dashed lines showing confidence intervals).

In our model we assumed that detection of a new case happens via two routes: either by contact tracing or by diagnosis because of symptoms (which are regulated by the parameters q and $\delta_{I}$, respectively). It is interesting to study how these two detection routes affect the spread of the epidemic. Among the infected individuals who escape contact tracing and are symptomatic, a fraction $\delta_{I}/(\delta_{I}+\alpha +\gamma_{I})$ are diagnosed. Figure 11 shows how this proportion, together with the proportion of individuals detected by contact tracing, affect the value of $R_{c}$. No substantial difference in the contribution of the two monitoring approaches is evident from the plot, however they have both relevant impacts in terms of containment strategies.

**Figure 12:**
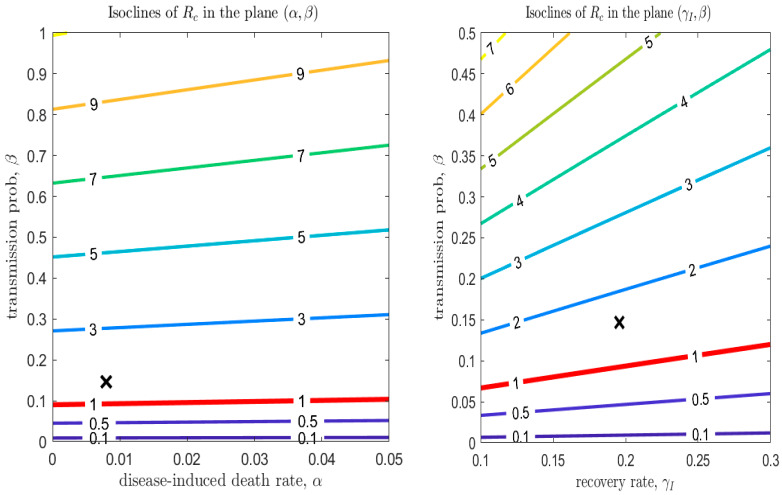
Left panel: values of $R_{c}$ in the plane (α,β). Right panel: values of $R_{c}$ in the plane $\left(\gamma_{I},\beta \right)$. The other parameters are those estimated on March 24. The red line corresponds to $R_{c}=1$ (dashed lines showing confidence intervals).

Mutation of the virus can affect some parameters in the transmission model, specifically the transmission probability, β, the disease-induced death rate, α, and the recovery rates. Figure 12 (left panel) shows that the modification of α has only a minor effect on the epidemic dynamics. However, the effect of a mutation in the recovery rate $\gamma_{I}$ is larger in magnitude (this can be observed explicitly in the equation for $R_{c}$, considering that $\gamma_{I}$ has a much larger scale than α). Although its effect on $R_{c}$ is minor compared to a reduction in the transmission probability (see Figure 12, right panel), the recovery rate is affected not only by mutations in the virulence of the pathogen, but also by improvements in treatment of the disease. Finally, Figure 7, Figure 8 and Figure 9 show that even a small reduction in the transmission probability can have a great impact in reducing the value of $R_{c}$: this emphasizes both the risks of possible mutations of the virus, but also the importance of use of personal protective equipment during the de-escalation phases.

## 4. Discussion

While the effects of interventions and protocols on the disease transmission dynamics and reproduction number can be quantified, there are additional considerations to be made in terms of the feasibility of the intervention package. For instance, the testing capacity in Ontario has been expanded and may continue to be utilized for the province to maintain the synergistic effects of speedy diagnosis, subsequent contact tracing, and isolation. Similarly, efforts have been made to enhance the contact tracing capacity in Ontario, including volunteer efforts, and may be maintained or heightened during de-escalation. Sustaining these key elements of the intervention package may allow for distancing measures to be relaxed, to allow resumption of economic activities. In future works, the model and analyses presented here can be expanded to include age-specific model parameters, including heterogeneity in the social contact rate, susceptibility and testing rate. In this light, an age-structured transmission model may be used to consider a wider range of de-escalation strategies with age-specific and setting-specific components. A variety of scenarios can be captured by utilizing setting-specific mixing contact matrices in the school, workplace, household, and community, including allowing a certain proportion of the workforce in some age groups, or specific age groups to resume activities such as attending schools or day care [10,11]. For a rotating working force strategy in the de-escalation phase one, sub-groups can be modeled for people with different working schedules to evaluate the efficiency of this strategy in re-opening workplaces to avoid further economic loss while controlling the reproduction number through contact reduction.

Through the parametrization and simulation of disease transmission models incorporating the interventions implemented in the province of Ontario, our work utilizes a general framework for intervention (escalation and de-escalation) evaluation and intervention scenario analysis. This study highlights the opportunity for evaluation of control measures and trends in disease transmission to inform the real-time and future decision-making for de-escalation, management, as well as risk assessment of COVID-19. Further, this study establishes a systematic model-fitting, analysis, and interpretation procedure for de-escalation and may be used as a reference for other regions.

## 5. Conclusions

We proposed a de-escalation strategy that simply reverses the escalation process that most Canadian provinces and territories, and especially, Ontario, have gone through. We used a transmission dynamics model fitted to the cumulative cases time series data to estimate effectiveness of interventions in terms of contact rate, probability of transmission per contact, detection rate, and proportion of isolated contacts. We used the parameters estimated during the escalation of interventions as baseline parameters for de-escalation scenario analysis. In particular, we explored how enhanced social distancing, personal protection, contact tracing, quarantine/isolation strength and diagnosis capacity can act synergistically to maintain the effective reproduction number below the threshold of 1, hence avoiding a rebound in infection. Our analysis can be used to inform decision makers on what interventions are strategically more convenient to invest in, for a successful containment of the disease during de-escalation.

## Figures and Tables

**Table 1:** School closure, emergency and public health emergency declarations in Canada by province and territories.

Province	School Closure	Date of Emergency Declaration and Type	Date of Closure of Non-Essential Establishments
British Columbia	14-March-2020	18-March-2020 (public health emergency)	21-March-2020
Alberta	14-March-2020	17-March-2020 (provincial public health emergency)	27-March-2020
Saskatchewan	21-March-2020	18-March-2020 (provincial state of emergency)	20-March-2020; Expanded 23-March-2020
Manitoba	14-March-2020	20-March-2020 (provincial state of emergency)	20-March-2020
Ontario	14-March-2020	17-March-2020 (provincial state of emergency)	23-March-2020; Expanded 25-Mar-2020
Quebec	14-March-2020	13-March-2020 (provincial public health emergency)	15-March-2020; Expanded 23-March-2020; Expanded further 28-March-2020
Newfoundland & Labrador	14-March-2020	18-March-2020 (public health emergency)	18-March-2020; Expanded 23-March-2020:
New Brunswick	14-March-2020	19-March-2020 (provincial state of emergency)	19-March-2020
Nova Scotia	14-March-2020	22-March-2020 (provincial state of emergency)	24-March-2020
Prince Edward Island	14-March-2020	16-March-2020 (public health emergency)	18-March-2020
Yukon	14-March-2020	18-March-2020 (public health emergency)	
Northwest Territories	14-March-2020	19-March-2020 (public health emergency)	
Nunavut	14-March-2020	19-March-2020 (public health emergency)	

**Table 2:** Parameter estimates for COVID-19 in Ontario, Canada.

**Parameter**		**Definitions**	**Mean (Std)**	**Source**
$c_{0}$		Contact rate before March 14	11.5801 (0.3456)	Estimated
$c_{1}$		Contact rate between March 14 to March 18	10.1202 (0.9185)	Estimated
$c_{2}$		Contact rate between March 18 to March 24	8.0495 (0.2787)	Estimated
c(t)	$c_{2}$	Constant contact rate at March 24	8.0495 (0.2787)	Estimated
	$r_{1}$	Exponential decrease in contact rate	0.0466 (0.0152)	Estimated
	$c_{b}$	Minimum contact rate after March 24	2.1987 (0.2400)	Estimated
β		Probability of transmission per contact	0.1469 (0.0023)	Estimated
$q_{0}$		Fraction of quarantined exposed individuals before March 24	0.1145 (0.0114)	Estimated
q(t)	$q_{0}$	Quarantine fraction at March 24	0.1145 (0.0114)	Estimated
	$r_{2}$	Exponential increase in quarantine fraction	0.1230 (0.0123)	Estimated
	$q_{b}$	The maximum quarantine fraction	0.3721 (0.0371)	Estimated
σ		Transition rate of exposed individuals to the infected class	1/5	[9]
λ		Rate at which the quarantined uninfected contacts were released into the wider community	1/14	[7]
ϱ		Probability of having symptoms among infected individuals	0.7036 (0.0261)	Estimated
$\delta_{I}$		Transition rate of symptomatic infected individuals to the quarantined infected class	0.1344 (0.0134)	Estimated
$\delta_{q}$		Transition rate of quarantined exposed individuals to the quarantined infected class	0.1237 (0.0086)	Estimated
$\gamma_{I}$		Recovery rate of symptomatic infected individuals	0.1957 (0.0111)	Estimated
$\gamma_{A}$		Recovery rate of asymptomatic infected individuals	0.139	[7]
$\gamma_{D}$		Recovery rate of quarantined diagnosed individuals	0.2	[8]
α		Disease-induced death rate	0.008	[8]
θ		Modification factor of asymptomatic infectiousness	0.0275 (0.0128)	Estimated
**Initial values**		**Definitions**	**Mean (Std)**	**Source**
S(0)		Initial susceptible population	$1.471\times 10^{7}$	Data
E(0)		Initial exposed population	8.9743 (0.6558)	Estimated
I(0)		Initial symptomatic infected population	5.3887 (0.9442)	Estimated
A(0)		Initial asymptomatic infected population	19.4186 (3.9406)	Estimated
$S_{q}\left(0\right)$		Initial quarantined susceptible population	0	Data
$E_{q}\left(0\right)$		Initial quarantined exposed population	0	Data
D(0)		Initial quarantined diagnosed population	5	Data
R(0)		Initial recovered population	0	Data

## Supplementary Materials

The following are available online at https://www.mdpi.com/2079-7737/9/5/100/s1, S1: Matlab code for parameter estimation (least square method).
