# Supplementary material for: De-Escalation by Reversing the Escalation with a Stronger Synergistic Package of Contact Tracing, Quarantine, Isolation and Personal Protection: Feasibility of Preventing a COVID-19 Rebound in Ontario, Canada, as a Case Study
Source: Biology (Basel). 2020 May 16;9(5):100. doi: 10.3390/biology9050100 (PMC7284446; doi:10.3390/biology9050100)
Supplement: Supplementary file 1 [file biology-09-00100-s001.pdf]

Supplementary material to: “De-escalation by Reversing the Escalation with a Stronger Synergistic Package of Contact Tracing, Quarantine, Isolation and Personal Protection: Feasibility of Preventing a COVDI-19 Rebound in Ontario, Canada, as a Case Study”

1. Matlab code for parameter estimation (least square method)

```
function    Parest_ON
clear all
clc

Dat_leiji=[5 6 8 11 15 15 20 20 22 28 28 32 35 37 42 59 79 103 145 177 189 214 258 ...
318 377 425 503 588 688 858 993 1144 1355 1706 1966 2392 2793 3255 3630 4038 4347 ...
4726 5276 5759 6237 6648 7049 7470 7953 8447 8961 9525 10010 10578 11184 11735];

AA=[length(Dat_leiji)];

for i=1:1

Num1=1;Num2=AA(i);
data1=zeros(3,length(Dat_leiji(Num1:Num2)));
data1(1,:)=0:length(Dat_leiji(Num1:Num2))-1;
data1(2,:)=Dat_leiji(Num1:Num2);
%data1(3,:)=Dat_death(Num1:Num2);
data.ydata=[data1'];


sigma=1/5;lambda=1/14;gam_A=0.139;

H0=5;
R0=0;
Sq0=0;
Eq0=0;

S0=1.471*10^7;
gam_H=0.2;
alpha=0.008;

par2=[sigma lambda gam_A Sq0 H0 R0 Eq0 S0 gam_H alpha];

par1guess = [0.15    12       0.12      0.7    0.15         1/7         1/8        10      5      20
0.03       9      8     0.1     2     0.1      0.35];
lb=[0.14                 11        0.1        0.6       0.1        1/10      1/10       1       1       10
0.01       9      7     0.01   2     0.01     0.3]; %6, 7, 9
ub=[0.15                  12        0.14       0.8       0.2        1/5       1/7        15      20     20
0.05       10     9     0.8     3     0.5       0.4];
```

```
options=optimset('Display','final','MaxIter', 20000, 'MaxFunEvals',1000000,'TolX',1e-6,'TolCon',1e-6,'Tolfun',1e-6);
[par1,fval] = fmincon(@kuzdist,par1guess,[],[],[],[],lb,ub,[],options,data1,par2)
%[par1,fval] = lsqnonlin(@kuzdist,par1guess,lb,ub,options,data_1,par2)
tvec_1 = [0:0.1:length(Dat_leiji)-1+80]; %go one time step beyond the data time

save par1 'par1'
par1;

beta=par1(1)
c0=par1(2)
q0=par1(3)
rho=par1(4)

gam_I=par1(5)
delta_I=par1(6)
delta_q=par1(7)

E0=par1(8)
I0=par1(9)
A0=par1(10)

theta=par1(11)
c1=par1(12)
c2=par1(13)
r1=par1(14)
cb=par1(15)
r2=par1(16)
qb=par1(17)

sigma=par2(1);
lambda=par2(2);
gam_A=par2(3);

Sq0=par2(4);
H0=par2(5);
R0=par2(6);
Eq0=par2(7);
S0=par2(8);
gam_H=par2(9);
alpha=par2(10);

save parameters_fit

X0=[S0 E0 I0 A0 Sq0 Eq0 H0 R0 H0 0];

[T1,X1]=systemsoln(par1,X0,tvec_1,par2);
```

```
for tn=0:1:length(Dat_leiji)-1
    i=tn+1;
    if tn<=17
    c(i)=c0;
elseif tn>17&tn<=21
    c(i)=c1;
elseif tn>21&tn<=27
    c(i)=c2;
else
    c(i)=(c2-cb)*exp(-r1*(tn-27))+cb;
end

if tn<=27
    q(i)=q0;
else
    q(i)=(q0-qb)*exp(-r2*(tn-27))+qb;
end

end

figure(2)
subplot(2,2,1)
plot(tvec_1,X1(:,9),'b-',data1(1,:),Dat_leiji(data1(1,:)+1),'bo','MarkerSize',4);
ylabel('Accumulated report cases')
title('(A)')

set(gca,'XTick',[0:10:140])
set(gca,'xticklabel',{'2/26','3/7','3/17','3/27','4/6','4/16','4/26','5/6','5/16','5/26','6/05','6/15','6/25','7/05','7/
15'});

subplot(222)
plot(tvec_1,X1(:,3),'-b')
hold on
ylabel('I(t)')
title('(B)')

set(gca,'XTick',[0:10:130])
set(gca,'xticklabel',{'2/26','3/7','3/17','3/27','4/6','4/16','4/26','5/6','5/16','5/26','6/05','6/15','6/25','7/05'});

subplot(223)
plot(tvec_1,X1(:,4),'-b')
hold on
title('(C)')
ylabel('A(t)')

set(gca,'XTick',[0:10:130])
set(gca,'xticklabel',{'2/26','3/7','3/17','3/27','4/6','4/16','4/26','5/6','5/16','5/26','6/05','6/15','6/25','7/05'});
```

```
subplot(224)
plot(tvec_1,X1(:,7),'-b')
hold on
title('(D)')
ylabel('D(t)')

set(gca,'XTick',[0:10:130])
set(gca,'xticklabel',{'2/26','3/7','3/17','3/27','4/6','4/16','4/26','5/6','5/16','5/26','6/05','6/15','6/25','7/05'});
end


function xdot=kuzode(tn,x,par1,par2)
beta=par1(1);
c0=par1(2);
q0=par1(3);
rho=par1(4);

gam_I=par1(5);
delta_I=par1(6);
delta_q=par1(7);

E0=par1(8);
I0=par1(9);
A0=par1(10);

theta=par1(11);
c1=par1(12);
c2=par1(13);
r1=par1(14);
cb=par1(15);
r2=par1(16);
qb=par1(17);

sigma=par2(1);
lambda=par2(2);
gam_A=par2(3);

Sq0=par2(4);
H0=par2(5);
R0=par2(6);
Eq0=par2(7);
S0=par2(8);
gam_H=par2(9);
alpha=par2(10);

X0=[S0 E0 I0 A0 Sq0 Eq0 H0 R0 H0 0];

if tn<=17
```

```
    c=c0;
elseif tn>17&tn<=21
    c=c1;
elseif tn>21&tn<=27
    c=c2;
else
    c=(c2-cb)*exp(-r1*(tn-27))+cb;
end

if tn<=27
    q=q0;
else
    q=(q0-qb)*exp(-r2*(tn-27))+qb;
end


x1=x(1);x2=x(2);x3=x(3);x4=x(4);x5=x(5);x6=x(6);x7=x(7);x8=x(8);
N=x1+x2+x3+x4+x5+x6+x7+x8;

xdot=[-(beta*c+c*q*(1-beta))*x1*(x3+theta*x4)/N+lambda*x5;
      beta*c*(1-q)*x1*(x3+theta*x4)/N-sigma*x2;
      sigma*rho*x2-(delta_I+alpha+gam_I)*x3;
      sigma*(1-rho)*x2-gam_A*x4;
      (1-beta)*c*q*x1*(x3+theta*x4)/N-lambda*x5;
      beta*c*q*x1*(x3+theta*x4)/N-delta_q*x6;
      delta_I*x3+delta_q*x6-(alpha+gam_H)*x7;
      gam_I*x3+gam_A*x4+gam_H*x7;
      delta_I*x3+delta_q*x6;
      alpha*x7;];

function d = kuzdist(par1,data1,par2)
E0=par1(8);
I0=par1(9);
A0=par1(10);

Sq0=par2(4);
H0=par2(5);
R0=par2(6);
Eq0=par2(7);
S0=par2(8);

X0=[S0 E0 I0 A0 Sq0 Eq0 H0 R0 H0 0];

d=0; % initialize sum of squares to zero

% solve the ode numerically at the given times
[T,x] = systemsoln(par1,X0,[0:1:data1(1,end)],par2);
% add to sum of squares
```

```
d = d + norm(x(:,9)' - data1(2,:))^2;

function [T,x]=systemsoln(par1,x0,tn,par2)
[T,x]=ode45(@kuzode,tn,x0,[],par1, par2);
```

2. Matlab code for computation of Rc and contour plots in two-parameter planes

```
% compute_Rc.m
% script to compute Rc in parameter planes from the transmission model used
% in
% Tang et al, "De-escalation by Reversing the Escalation with a Stronger
% Synergistic Package of Contact Tracing, Quarantine, Isolation and
% Personal Protection: Feasibility of Preventing a COVID-19 Rebound in
% Ontario, Canada, as a Case Study", Biology, 2020.
%

clear

%% Load parameters

alpha=0.008;
gammaA=0.139;

% Mean values from bootstrap samples
% load('X') % contains the bootstrap samples
% KK=mean(X);
beta0 = 0.1469; % KK(1);
c0 = 11.5801; % KK(2);
q0 = 0.1145; % KK(3);
rho = 0.7036; % KK(4);
gammaI = 0.1957; % KK(5);
deltaI = 0.1344; % KK(6);
deltaq = 0.1237; % KK(7);
% E0_m = 8.9742; % KK(8)
% I0_m = 5.3887; % KK(9)
% A0_m = 19.4186; % KK(10)
theta = 0.0275; % KK(11);
c1 = 10.1202; % KK(12);
c2 = 8.0495; % KK(13);
r1 = 0.0466; % KK(14);
cb = 2.1987; % KK(15);
r2 = 0.1230; % KK(16);
qb = 0.3721; % KK(17);

% Standard deviations
% KK1=std(X);
```

```
beta0_s = 0.0023; % KK1(1);
c0_s = 0.345; % KK1(2);
q0_s = 0.0114; % KK1(3);
rho_s = 0.0261; % KK1(4);
gammaI_s = 0.0111; % KK1(5);
deltaI_s = 0.0134; % KK1(6);
deltaq_s = 0.0086; % KK1(7);
% E0_s = 0.6558; % KK1(8)
% I0_s = 0.9442; % KK1(9)
% A0_s = 3.9406; % KK1(10)
theta_s = 0.0128; % KK1(11);
c1_s = 0.9158; % KK1(12);
c2_s = 0.2787; % KK1(13);
r1_s = 0.0152; % KK1(14);
cb_s = 0.2400; % KK1(15);
r2_s = 0.0123; % KK1(16);
qb_s = 0.0371; % KK1(17);

% Rc = @(beta,q,deltaI) rho*(beta.*c.*(1-q))./(deltaI+alpha+gammaI) ...
%         + (1-rho)*(beta.*c.*(1-q))*theta/gammaA;

%
min_q = q0;
max_q = qb;

min_c=cb;
max_c=c2;

period_index = 3; % 1: before March 14; 2: March 14-18; 3: after March 24

switch period_index
    case 1
        % before March 14
        cconst=c0;
        cconst_s=c0_s;
        period = 'with parameters before March 14';
        nameper = 'March14';
    case 2
        % between March 14-18
        cconst=c1;
        cconst_s=c1_s;
        period = 'with parameters as in March 14-18';
        nameper = 'March14-18';
    case 3
        % between March 18-24
        cconst=c2;
        cconst_s=c2_s;
        period = 'with parameters at March 24';
```

```
        nameper = 'March24';
end

red_coeff = [1,0.75,0.5,0.25]; % vector of reductions in contact rate

%% In terms of quarantine and time to diagnosis

figure(11); clf

for ind_red=1:length(red_coeff)

    red_cont = red_coeff(ind_red);
    c=red_cont*cconst;

    % Countour plot
    xx=linspace(0,1,100); Lx=length(xx); % quarantine fraction
    yy=linspace(2,10,100); Ly = length(yy); % time for diagnosis
    [XX,YY]=meshgrid(xx,yy);

    Rc_matrix = rho*(beta0.*c.*(1-XX))./(1./YY+alpha+gammaI) ...
        + (1-rho)*(beta0.*c*(1-XX))*theta/gammaA;

    figure(11)
    subplot(2,2,ind_red)
    contour(XX,YY,Rc_matrix,[0,0.1,0.2:0.2:0.8,1.5:0.5:4],'ShowText','on','LineWidth',2); hold on
    contour(XX,YY,Rc_matrix,[1 1],'ShowText','on','EdgeColor','red','LineWidth',3); hold on
    axis([xx(1) xx(end) yy(1) yy(end)])

    plot([min_q,max_q],[1/deltaI,1/deltaI],'k-x','MarkerSize',10,'LineWidth',2);
    xlabel('quarantine fraction, $q$','Interpreter','latex'); ylabel('time for diagnosis,
$1/\delta_I$','Interpreter','latex');
    title(['Contact reduced by ',num2str((1-red_cont)*100),'\%'],'Interpreter','latex');
    sgt = sgtitle(['Isoclines of $R_c$ in the plane $(q,1/\delta_I)$, ',period],'Interpreter','latex'); %,'
and contact rate ',num2str(c0)]}
    sgt.FontSize = 10;

end

set(gcf, 'Position',[360 140 640 460])
savefig(['Rc_',nameper,'_q_timediag'])
saveas(gcf,['Rc_',nameper,'_q_timediag.png'])

%% In terms of fraction of quarantine and symptomatic
% (who escaped contact tracing) who are detected by diagnosis

figure(17); clf
clear Rc_matrix
```

```
for ind_red=1:length(red_coeff)

    red_cont = red_coeff(ind_red);
    c=red_cont*cconst;

    % Countour plot
    xx=0.3:0.1:0.8; % linspace(0,1,100);
    Lx=length(xx); % quarantine fraction
    yy=0.3:0.1:0.8; % linspace(0,1,100);
    Ly = length(yy); % fraction of symptomatic who are detected

    % diagnosis rate
    [XX,YY]=meshgrid(xx,yy);

    Delta_I = YY./(1-YY)*(alpha+gammaI);

    R0_matrix = rho*(beta0.*c.*(1-XX))./(Delta_I+alpha+gammaI) ...
        + (1-rho)*(beta0.*c*(1-XX))*theta/gammaA;

    figure(17)
    subplot(2,2,ind_red)
    contour(XX,YY,R0_matrix,[0,0.1,0.2:0.2:0.8,1.5:0.5:4],'ShowText','on','LineWidth',2); hold on
    contour(XX,YY,R0_matrix,[1 1],'ShowText','on','EdgeColor','red','LineWidth',3); hold on
%     plot(Q0_line,yy,'k');
%     axis([xx(1) xx(end) yy(1) yy(end)])

%     plot([min_q,max_q],[1/deltaI,1/deltaI],'-x','MarkerSize',10,'LineWidth',2);
    xlabel('quarantine fraction','Interpreter','latex'); ylabel('fraction of
diagnosed','Interpreter','latex');
    title(['Contact reduced by ',num2str((1-red_cont)*100),'\%'],'Interpreter','latex');
    sgt = sgtitle(['Isoclines of $R_c$ in the plane $(q, \frac{\delta_I}{\delta_I+\alpha
+\gamma_I})$, ',period],'Interpreter','latex'); %,' and contact rate ',num2str(c0)]}

end

set(gcf, 'Position',[360 140 640 460])
savefig(['Rc_',nameper,'_q_DetSympt'])
saveas(gcf,['Rc_',nameper,'_q_DetSympt.png'])


%% In terms of quarantine and transmission probability % note that they always appear as
beta*(1-q)

figure(12); clf
clear Rc_matrix

for ind_red=1:length(red_coeff)
```

```
    red_cont = red_coeff(ind_red);
    c=red_cont*cconst;

    % Countour plot
    xx=linspace(0,1,100); Lx=length(xx); % quarantine fraction
    yy=linspace(0,1,100); Ly = length(yy); % transmission probability
    [XX,YY]=meshgrid(xx,yy);

    Rc_matrix = rho*(YY.*c.*(1-XX))./(deltaI+alpha+gammaI) ...
        + (1-rho)*(YY.*c.*(1-XX))*theta/gammaA;

    figure(12)
    subplot(2,2,ind_red)
    contour(XX,YY,Rc_matrix,[0.1,0.5,1:2:20],'ShowText','on','LineWidth',2); hold on
    contour(XX,YY,Rc_matrix,[1 1],'ShowText','on','EdgeColor','red','LineWidth',3); hold on
    plot([min_q,max_q],[deltaI,deltaI],'k-x','MarkerSize',10,'LineWidth',2);
    xlabel('quarantine fraction, $q$','Interpreter','latex'); ylabel('transmission prob,
$\beta$','Interpreter','latex');
    title(['Contact reduced by ',num2str((1-red_cont)*100),'\%'],'Interpreter','latex');
    sgt = sgtitle(['Isoclines of $R_c$ in the plane $(q,\beta)$, ',period],'Interpreter','latex'); %,' and
contact rate ',num2str(c0)]}
    sgt.FontSize = 10;
end

set(gcf, 'Position',[360 140 640 460])
savefig(['Rc_',nameper,'_q_beta'])
saveas(gcf,['Rc_',nameper,'_q_beta.png'])

%% In terms of death rate and transmission probability % mutations
figure(18); clf

clear Rc_matrix
contact = c2; %[cb, c2, c1, c0];

for ind_c=1:length(contact)

    c=contact(ind_c);

    % Countour plot
    xx=linspace(0,0.05,100); Lx=length(xx); % disease-induced death rate
    yy=linspace(0,1,100); Ly = length(yy); % transmission probability
    [XX,YY]=meshgrid(xx,yy);

    Rc_matrix = rho*(YY.*c.*(1-qb))./(deltaI+XX+gammaI) ...
        + (1-rho)*(YY.*c.*(1-qb))*theta/gammaA;

    figure(18)
%   subplot(2,2,ind_c)
```

```
    contour(XX,YY,Rc_matrix,[0.1,0.5,1:2:20],'ShowText','on','LineWidth',2); hold on
    contour(XX,YY,Rc_matrix,[1 1],'ShowText','on','EdgeColor','red','LineWidth',3); hold on
    plot(alpha,beta0,'k-x','MarkerSize',10,'LineWidth',2);
    xlabel('disease-induced death rate, $\alpha$','Interpreter','latex'); ylabel('transmission prob,
$\beta$','Interpreter','latex');
%     title(['Average ',num2str(c),' contacts per day'],'Interpreter','latex');
    sgt = sgtitle(['Isoclines of $R_c$ in the plane $(\alpha,\beta)$'],'Interpreter','latex'); %,' and
contact rate ',num2str(c0)]}
    sgt.FontSize = 10;
end

% set(gcf, 'Position',[360 140 640 460])
set(gcf, 'Position',[360 140 400 400])
savefig(['Rc_',nameper,'_alpha_beta'])
saveas(gcf,['Rc_',nameper,'_alpha_beta.png'])

%% In terms of recovery rate and transmission probability % mutations
figure(19); clf

clear Rc_matrix
contact = c2; %[cb, c2, c1, c0];

for ind_c=1:length(contact)
    c=contact(ind_c);

    % Countour plot
    xx=linspace(0.1,0.3,100); Lx=length(xx); % recovery rate gammaI
    yy=linspace(0,0.5,100); Ly = length(yy); % transmission probability
    [XX,YY]=meshgrid(xx,yy);

    Rc_matrix = rho*(YY.*c.*(1-qb))./(deltaI+alpha+XX) ...
        + (1-rho)*(YY.*c.*(1-qb))*theta/gammaA;

    figure(19)
%     subplot(2,2,ind_c)
    if ind_c==1
        contour(XX,YY,Rc_matrix,[0.1,0.5,1:1:20],'ShowText','on','LineWidth',2); hold on
    else
        contour(XX,YY,Rc_matrix,[0.1,0.5,1:2:20],'ShowText','on','LineWidth',2); hold on
    end
    contour(XX,YY,Rc_matrix,[1 1],'ShowText','on','EdgeColor','red','LineWidth',3); hold on
    plot(gammaI,beta0,'k-x','MarkerSize',10,'LineWidth',2);
    xlabel('recovery rate, $\gamma_I$','Interpreter','latex'); ylabel('transmission prob,
$\beta$','Interpreter','latex');
%     title(['Average ',num2str(c),' contacts per day'],'Interpreter','latex');
    sgt = sgtitle(['Isoclines of $R_c$ in the plane $(\gamma_I,\beta)$'],'Interpreter','latex'); %,'
and contact rate ',num2str(c0)]}
    sgt.FontSize = 10;
```

```
end

% set(gcf, 'Position',[360 140 640 460])
set(gcf, 'Position',[360 140 400 400])
savefig(['Rc_',nameper,'_gammaI_beta'])
saveas(gcf,['Rc_',nameper,'_gammaI_beta.png'])

%% In terms of quarantine and contact rate

figure(13); clf

time_diag = 10:-1:5; % values of delta_I for subplots

for ind_red=1:length(time_diag)

    delt = 1./time_diag(ind_red);

    % Countour plot
    xx=linspace(0,1,100); Lx=length(xx); % quarantine fraction
    yy=linspace(0,12,100); Ly=length(yy); % contact rate
    [XX,YY]=meshgrid(xx,yy);

    Rc_matrix = rho*(beta0.*YY.*(1-XX))./(delt+alpha+gammaI) ...
        + (1-rho)*(beta0.*YY.*(1-XX))*theta/gammaA;

    figure(13)
    subplot(3,2,ind_red)
    contour(XX,YY,Rc_matrix,[0.1,0.5,1:0.5:3],'ShowText','on','LineWidth',2); hold on
    contour(XX,YY,Rc_matrix,[1 1],'ShowText','on','EdgeColor','red','LineWidth',3); hold on
%    plot([min_q,max_q],[max_c,min_c],'k-x','MarkerSize',10,'LineWidth',2);
    xlabel('quarantine fraction, $q$','Interpreter','latex'); ylabel('contacts per day,
$c$','Interpreter','latex');
    title([num2str(1/delt),' days diagnosis time'],'Interpreter','latex');
    sgt = sgtitle({'Isoclines of $R_c$ in the plane $(q,c)$'},'Interpreter','latex');
    sgt.FontSize = 10;
end

%set(gcf, 'Position',[360 198 560 840])
set(gcf, 'Position',[360 10 640 840])
% savefig(['Rc_q_contact'])
% saveas(gcf,['Rc_q_contact.png'])



%%%%%%%%%%%%%%%%%%%%%%%%%%%%%%%%%%%%%%%%%%
%% CI for Rc from bootstrap realizations

%load 'X'
```

```
% par = [rho,theta,gammaI,beta,deltaI,q0,c2];
Sam_Par=[X(:,4),X(:,11),X(:,5),X(:,1),X(:,6),X(:,3),X(:,13)];

Rc_samples = Sam_Par(:,1).*(Sam_Par(:,4).*Sam_Par(:,7).*(1-Sam_Par(:,6)))./...
       (Sam_Par(:,5)+alpha+Sam_Par(:,3)) + (1-Sam_Par(:,1)).*...
       (Sam_Par(:,4).*Sam_Par(:,7).*(1-Sam_Par(:,6))).*Sam_Par(:,2)/gammaA;

%% As a function of time

% par = [rho,theta,gammaI,beta,deltaI,q,c];
Sam_Par=[X(:,4),X(:,11),X(:,5),X(:,1),X(:,6),X(:,3),X(:,13)];

t_grid0=0:17;
Rc_mean_vec0=zeros(length(t_grid0),1);
Rc_sd_vec0=zeros(length(t_grid0),1);
for ii=1:length(t_grid0)

       Sam_Par(:,7)=X(:,2); % (c0) time-varying c
       Sam_Par(:,6)=X(:,3); % time-varying q

       Rc_samples = Sam_Par(:,1).*(Sam_Par(:,4).*Sam_Par(:,7).*(1-Sam_Par(:,6)))./...
       (Sam_Par(:,5)+alpha+Sam_Par(:,3)) + (1-Sam_Par(:,1)).*...
       (Sam_Par(:,4).*Sam_Par(:,7).*(1-Sam_Par(:,6))).*Sam_Par(:,2)/gammaA;

       Rc_mean_vec0(ii)=mean(Rc_samples);
       Rc_sd_vec0(ii) = std(Rc_samples);
end
CI_l0=Rc_mean_vec0-1.96*Rc_sd_vec0;
CI_u0=Rc_mean_vec0+1.96*Rc_sd_vec0;

figure(5); clf
plot([0,100],[1,1],'Color', [10 10 10]/255); hold on

plot(t_grid0,Rc_mean_vec0,'b-','LineWidth',2); hold on
plot(t_grid0,CI_l0,'b--','LineWidth',1);
plot(t_grid0,CI_u0,'b--','LineWidth',1);
hold on

t_grid1=17:21;
Rc_mean_vec1=zeros(length(t_grid1),1);
Rc_sd_vec1=zeros(length(t_grid1),1);
for ii=1:length(t_grid1)
       Sam_Par(:,7)=X(:,12); % (c1) time-varying c
       Sam_Par(:,6)=X(:,3); % time-varying q

       Rc_samples = Sam_Par(:,1).*(Sam_Par(:,4).*Sam_Par(:,7).*(1-Sam_Par(:,6)))./...
       (Sam_Par(:,5)+alpha+Sam_Par(:,3)) + (1-Sam_Par(:,1)).*...
```

```
    (Sam_Par(:,4).*Sam_Par(:,7).*(1-Sam_Par(:,6))).*Sam_Par(:,2)/gammaA;

    Rc_mean_vec1(ii)=mean(Rc_samples);
    Rc_sd_vec1(ii) = std(Rc_samples);
end
CI_l1=Rc_mean_vec1-1.96*Rc_sd_vec1;
CI_u1=Rc_mean_vec1+1.96*Rc_sd_vec1;

%figure(5);
plot(t_grid1,Rc_mean_vec1,'b-','LineWidth',2); hold on
plot(t_grid1,CI_l1,'b--','LineWidth',1)
plot(t_grid1,CI_u1,'b--','LineWidth',1)
hold on


t_grid2=21:27;
Rc_mean_vec2=zeros(length(t_grid2),1);
Rc_sd_vec2=zeros(length(t_grid2),1);
for ii=1:length(t_grid2)

        Sam_Par(:,7)=X(:,13); % (c2) time-varying c
        Sam_Par(:,6)=X(:,3); % time-varying q

        Rc_samples = Sam_Par(:,1).*(Sam_Par(:,4).*Sam_Par(:,7).*(1-Sam_Par(:,6)))./...
        (Sam_Par(:,5)+alpha+Sam_Par(:,3)) + (1-Sam_Par(:,1)).*...
        (Sam_Par(:,4).*Sam_Par(:,7).*(1-Sam_Par(:,6))).*Sam_Par(:,2)/gammaA;

        Rc_mean_vec2(ii)=mean(Rc_samples);
        Rc_sd_vec2(ii) = std(Rc_samples);

end
CI_l2=Rc_mean_vec2-1.96*Rc_sd_vec2;
CI_u2=Rc_mean_vec2+1.96*Rc_sd_vec2;

%figure(5);
plot(t_grid2,Rc_mean_vec2,'b-','LineWidth',2); hold on
plot(t_grid2,CI_l2,'b--','LineWidth',1);
plot(t_grid2,CI_u2,'b--','LineWidth',1);
hold on

t_grid3=27:0.5:55;
Rc_mean_vec3=zeros(length(t_grid3),1);
Rc_sd_vec3=zeros(length(t_grid3),1);
for ii=1:length(t_grid3)

    Sam_Par(:,7)=(X(:,13)-X(:,15)).*exp(-X(:,14)*(t_grid3(ii)-27))+X(:,15); % exponential time-
varying c
    Sam_Par(:,6)=(X(:,3)-X(:,17)).*exp(-X(:,16)*(t_grid3(ii)-27))+X(:,17); % time-varying q
```

```
    Rc_samples = Sam_Par(:,1).*(Sam_Par(:,4).*Sam_Par(:,7).*(1-Sam_Par(:,6)))./...
    (Sam_Par(:,5)+alpha+Sam_Par(:,3)) + (1-Sam_Par(:,1)).*...
    (Sam_Par(:,4).*Sam_Par(:,7).*(1-Sam_Par(:,6))).*Sam_Par(:,2)/gammaA;

    Rc_mean_vec3(ii)=mean(Rc_samples);
    Rc_sd_vec3(ii) = std(Rc_samples);

end
CI_l3=Rc_mean_vec3-1.96*Rc_sd_vec3;
CI_u3=Rc_mean_vec3+1.96*Rc_sd_vec3;

%figure(5);
plot(t_grid3,Rc_mean_vec3,'b-','LineWidth',2); hold on
plot(t_grid3,CI_l3,'b--','LineWidth',1);
plot(t_grid3,CI_u3,'b--','LineWidth',1);
hold on

axis([0 t_grid3(end) 0.5 3.5])

%xlabel('days since Feb 26','Interpreter','latex');
ylabel('$R_t$','Interpreter','latex');
title('Time-varying reproduction number $R_t$','Interpreter','latex');


set(gca,'XTick',[0,4:7:130])
set(gca,'xticklabel',{'2/26','3/1','3/8','3/15','3/22','3/29','4/5','4/12','4/19'});
%xlabel('time')

savefig('Rt')
saveas(gcf,'Rt.png')


%% Plot of confidence intervals in parameters planes

figure(15); clf

Sam_Par=[X(:,4),X(:,11),X(:,5),X(:,1),X(:,6),X(:,3),X(:,13)];

    switch period_index
        case 1
            Sam_Par(:,7)=X(:,2); % (c0)
        case 2
            Sam_Par(:,7)=X(:,12); % (c1)
        case 3
            Sam_Par(:,7)=X(:,13); % (c2)
    end
```

```
% Countour plot
xx=linspace(0,1,100); Lx=length(xx); % quarantine fraction
yy=linspace(2,10,100); Ly = length(yy); % time for diagnosis

% diagnosis rate
[XX,YY]=meshgrid(xx,yy);

for ind_1 = 1:Lx
    for ind_2 = 1:Ly

        Sam_Par(:,6)=xx(ind_1)*ones(500,1); % quarantine fraction
        Sam_Par(:,5)=1./yy(ind_2)*ones(500,1); % diagnosis rate

        Rc_samples = Sam_Par(:,1).*(Sam_Par(:,4).*Sam_Par(:,7).*(1-Sam_Par(:,6)))./...
        (Sam_Par(:,5)+alpha+Sam_Par(:,3)) + (1-Sam_Par(:,1)).*...
        (Sam_Par(:,4).*Sam_Par(:,7).*(1-Sam_Par(:,6))).*Sam_Par(:,2)/gammaA;

        Rc_mean_mat(ind_2,ind_1)=mean(Rc_samples);
        Rc_sd_mat(ind_2,ind_1) = std(Rc_samples);
    end
end
CI_l2_mat=Rc_mean_mat-1.96*Rc_sd_mat;
CI_u2_mat=Rc_mean_mat+1.96*Rc_sd_mat;

figure(11)
subplot(2,2,1)
contour(XX,YY,CI_l2_mat,[1,1],'r--','ShowText','off','LineWidth',1); hold on
contour(XX,YY,CI_u2_mat,[1,1],'r--','ShowText','off','LineWidth',1); hold on
savefig(['Rc_',nameper,'_q_timediag'])
saveas(gcf,['Rc_',nameper,'_q_timediag.png'])

%%

% quarantine and transmission

Sam_Par=[X(:,4),X(:,11),X(:,5),X(:,1),X(:,6),X(:,3),X(:,13)];

    switch period_index
        case 1
            Sam_Par(:,7)=X(:,2); % (c0)
        case 2
            Sam_Par(:,7)=X(:,12); % (c1)
        case 3
            Sam_Par(:,7)=X(:,13); % (c2)
    end

% Countour plot
xx=linspace(0,1,100); Lx=length(xx); % quarantine fraction
```

```
yy=linspace(0,1,100); Ly = length(yy); % transmission probability

[XX,YY]=meshgrid(xx,yy);

clear Rc_mean_mat Rc_sd_mat
for ind_1 = 1:Lx
    for ind_2 = 1:Ly

        % par = [rho,theta,gammaI,beta,deltaI,q,c];
        Sam_Par(:,6)=xx(ind_1)*ones(500,1);
        Sam_Par(:,4)=yy(ind_2)*ones(500,1);

        Rc_samples = Sam_Par(:,1).*(Sam_Par(:,4).*Sam_Par(:,7).*(1-Sam_Par(:,6)))./...
        (Sam_Par(:,5)+alpha+Sam_Par(:,3)) + (1-Sam_Par(:,1)).*...
        (Sam_Par(:,4).*Sam_Par(:,7).*(1-Sam_Par(:,6))).*Sam_Par(:,2)/gammaA;

        Rc_mean_mat(ind_2,ind_1)=mean(Rc_samples);
        Rc_sd_mat(ind_2,ind_1) = std(Rc_samples);
    end
end
CI_l2_mat=Rc_mean_mat-1.96*Rc_sd_mat;
CI_u2_mat=Rc_mean_mat+1.96*Rc_sd_mat;

figure(12)
subplot(2,2,1)
contour(XX,YY,CI_l2_mat,[1,1],'r--','ShowText','off','LineWidth',1); hold on
contour(XX,YY,CI_u2_mat,[1,1],'r--','ShowText','off','LineWidth',1); hold on
savefig(['Rc_',nameper,'_q_beta'])
saveas(gcf,['Rc_',nameper,'_q_beta.png'])

% figure(15)
% subplot(1,3,2)
% contour(XX,YY,Rc_mean_mat,[1,1],'b','ShowText','on','LineWidth',2); hold on
% contour(XX,YY,CI_l2_mat,[1,1],'b--','ShowText','off','LineWidth',1); hold on
% contour(XX,YY,CI_u2_mat,[1,1],'b--','ShowText','off','LineWidth',1); hold on
% axis([xx(1) xx(end) yy(1) yy(end)])
% xlabel('quarantine fraction, $q$','Interpreter','latex'); ylabel('transmission probability,
$\beta$','Interpreter','latex');
% %title(['Contact reduced by ',num2str((1-red_cont)*100),'\%'],'Interpreter','latex');
% hold on

%% quarantine and contact

Sam_Par=[X(:,4),X(:,11),X(:,5),X(:,1),X(:,6),X(:,3),X(:,13)];
Sam_Par(:,7)=X(:,13); % (c2) time-varying c
Sam_Par(:,6)=X(:,3); % time-varying q

% Countour plot
```

```
xx=linspace(0,1,100); Lx=length(xx); % quarantine fraction
yy=linspace(0,12,100); Ly = length(yy); % contact rate

[XX,YY]=meshgrid(xx,yy);

clear Rc_mean_mat Rc_sd_mat

time_diag = 10:-1:5; % values of delta_I for subplots
for ind_red=1:length(time_diag)

    delt = 1./time_diag(ind_red);

for ind_1 = 1:Lx
    for ind_2 = 1:Ly

        % par = [rho,theta,gammaI,beta,deltaI,q,c];
        Sam_Par(:,6)=xx(ind_1)*ones(500,1);
        Sam_Par(:,7)=yy(ind_2)*ones(500,1);

        Sam_Par(:,5)=delt*ones(500,1); % we fix it at the first panel

        Rc_samples = Sam_Par(:,1).*(Sam_Par(:,4).*Sam_Par(:,7).*(1-Sam_Par(:,6)))./...
        (Sam_Par(:,5)+alpha+Sam_Par(:,3)) + (1-Sam_Par(:,1)).*...
        (Sam_Par(:,4).*Sam_Par(:,7).*(1-Sam_Par(:,6))).*Sam_Par(:,2)/gammaA;

        Rc_mean_mat(ind_2,ind_1)=mean(Rc_samples);
        Rc_sd_mat(ind_2,ind_1) = std(Rc_samples);
    end
end
CI_l2_mat=Rc_mean_mat-1.96*Rc_sd_mat;
CI_u2_mat=Rc_mean_mat+1.96*Rc_sd_mat;


figure(13)
subplot(3,2,ind_red)
contour(XX,YY,CI_l2_mat,[1,1],'r--','ShowText','off','LineWidth',1); hold on
contour(XX,YY,CI_u2_mat,[1,1],'r--','ShowText','off','LineWidth',1); hold on
savefig(['Rc_q_contact'])
saveas(gcf,['Rc_q_contact.png'])

end

%% fraction of quarantine and symptomatic
% (who escaped contact tracing) who are detected by diagnosis

Sam_Par=[X(:,4),X(:,11),X(:,5),X(:,1),X(:,6),X(:,3),X(:,13)];
Sam_Par(:,7)=X(:,13); % (c2) time-varying c
Sam_Par(:,6)=X(:,3); % time-varying q
```

```
% Countour plot
xx=0.3:0.1:0.8; Lx=length(xx); % quarantine fraction
yy=0.3:0.1:0.8; Ly = length(yy); % fraction of symptomatic who are detected

[XX,YY]=meshgrid(xx,yy);

clear Rc_mean_mat Rc_sd_mat

c=cconst;

for ind_1 = 1:Lx
    for ind_2 = 1:Ly

        % par = [rho,theta,gammaI,beta,deltaI,q,c];
        Sam_Par(:,6)=xx(ind_1)*ones(500,1);
        Sam_Par(:,5)=(yy(ind_2)./(1-yy(ind_2))*(alpha+gammaI))*ones(500,1);

        Rc_samples = Sam_Par(:,1).*(Sam_Par(:,4).*Sam_Par(:,7).*(1-Sam_Par(:,6)))./...
        (Sam_Par(:,5)+alpha+Sam_Par(:,3)) + (1-Sam_Par(:,1)).*...
        (Sam_Par(:,4).*Sam_Par(:,7).*(1-Sam_Par(:,6))).*Sam_Par(:,2)/gammaA;

        Rc_mean_mat(ind_2,ind_1)=mean(Rc_samples);
        Rc_sd_mat(ind_2,ind_1) = std(Rc_samples);
    end
end
CI_l2_mat=Rc_mean_mat-1.96*Rc_sd_mat;
CI_u2_mat=Rc_mean_mat+1.96*Rc_sd_mat;

figure(17)
subplot(2,2,1)
contour(XX,YY,CI_l2_mat,[1,1],'r--','ShowText','off','LineWidth',1); hold on
contour(XX,YY,CI_u2_mat,[1,1],'r--','ShowText','off','LineWidth',1); hold on
savefig(['Rc_',nameper,'_q_DetSympt'])
saveas(gcf,['Rc_',nameper,'_q_DetSympt.png'])
```
